# Measurement of the distance between tumor micro-foci and gross tumor in rectal cancer pathological specimens: implication on margin distance of clinical target volume treated with high-dose radiotherapy for rectal cancer

**DOI:** 10.1007/s10147-024-02582-4

**Published:** 2024-07-08

**Authors:** Xu-Jie Bao, Xiao-Yan Chen, Lu Wen, Yuan-Yuan Liu, En-Hao Yu, Zheng Wu, Ke Liu, Ju-Mei Zhou, Su-Yu Zhu

**Affiliations:** 1 Department of Radiation Oncology, Hunan Cancer Hospital and the Affiliated Cancer Hospital of Xiang Ya School of Medicine, Central South University, No. 582 Xianjiahu Rd., Yuelu District, Changsha, 410013 People’s Republic of China; 2 Department of Oncology, XiangYa ChangDe Hospital, Changde, Hunan People’s Republic of China; 3 Department of Pathology, Hunan Cancer Hospital and the Affiliated Cancer Hospital of Xiang Ya School of Medicine, Central South University, Changsha, People’s Republic of China; 4 Department of Diagnostic Radiology, Hunan Cancer Hospital and the Affiliated Cancer Hospital of Xiang Ya School of Medicine, Central South University, Changsha, People’s Republic of China; 5 Department of Radiation Oncology, The First Hospital of Hunan University of Chinese Medicine, Changsha, Hunan People’s Republic of China; 6 Qilu Hospital of Shandong University, Qingdao, People’s Republic of China

**Keywords:** Rectal adenocarcinoma, Tumor micro-focus, Pathological specimen, Margin distance of clinical target volume, High-dose radiotherapy

## Abstract

**Purpose:**

To measure the micro-foci distance away from gross tumor and to provide reference to create the clinical target volume (CTV) margin for boost radiotherapy in rectal adenocarcinoma.

**Methods:**

Twenty-eight rectal cancer surgical specimens of only total mesorectal excision were collected. The pathological specimens were retrospectively measured, and the nearest distance between the tumor micro-foci and gross tumor was microscopically measured. The “in vivo–in vitro” retraction factor was calculated as the ratio of the deepest thickness laterally and the vertical height superior/inferiorly of the rectal tumor measured in MRI and those measured in immediate pathological specimens. The retraction factor during pathological specimen processing was calculated as the distance ratio before and after dehydration in the lateral, superior, and inferior sides by the “knot marking method.” The distances of tumor micro-foci were individually corrected with these two retraction factors.

**Results:**

The mean “in vivo–in vitro” tumor retraction factors were 0.913 peripherally and 0.920 superior/inferiorly. The mean tumor specimen processing retraction factors were 0.804 peripherally, 0.815 inferiorly, and 0.789 superiorly. Of 28 patients, 14 cases (50.0%) had 24 lateral micro-foci, 8 cases (28.6%) had 13 inferior micro-foci, and 7 cases (25.0%) had 19 superior micro-foci. The 95th percentiles of the micro-foci distance for 28 patients were 6.44 mm (peripheral), 5.54 mm (inferior), and 5.42 mm (superior) after retraction correction.

**Conclusion:**

The micro-foci distances of 95% of rectal adenocarcinoma patients examined were within 6.44 mm peripherally, 5.54 mm inferiorly, and 5.42 mm superiorly. These findings provide reference to set the boost radiotherapy CTV margin for rectal cancer.

## Introduction

Recently, non-surgical management for locally advanced rectal cancer (LARC) after neoadjuvant chemoradiotherapy (NCRT) has attracted the interest of clinical researchers. Some researchers have suggested that “watch and wait” could be applied to the patients who achieved clinical complete remission (cCR) after neoadjuvant radiotherapy and chemotherapy, and that rescue surgery should only be conducted in patients with tumor regrowth. The curative effect of this approach is similar to that of the standard treatment. Therefore, 74–94.8% of patients can be exempted from surgery and the anal function can be retained, allowing a high quality of life to be maintained after treatment [1–3]. For those patients with good response to NCRT, local excision with sphincter preservation has also shown similar oncologic safety to total mesorectal excision (TME) [4].

The standard treatment for LARC is NCRT, followed by 6–8 weeks delayed TME. Chemoradiation consists of a total radiation dose of 46–50.4 Gy combined with oral capecitabine or infusional 5-fluororouracil. However, the pathological complete response (pCR) ratio was in the low range of 15–27% in large-scale clinical studies with chemoradiotherapy [3, 5]. Therefore, developing methods to improve the cCR rate by NCRT has become an area of active clinical research. Studies of intensified chemotherapy of NCRT did not show survival benefit, and with more toxicity for LARC [6, 7]. Several studies with higher than 50.4 Gy escalated radiation doses resulted in higher cCR rates and had the potential to improve long-term outcomes for LARC patients [8]. Meta-analysis showed that there was a linear relationship between cCR rate and local radiotherapy dose for rectal cancer [9]. However, local high-dose radiotherapy is always limited by the radiotherapy complications of normal tissues around the tumor because of receiving a higher radiation dose. Therefore, accurate determination of the clinical target volume (CTV) margin distances (distances between tumor micro-foci and the gross tumor) is essential for the success of dose escalation radiotherapy. Surgical pathological specimens are considered the “gold standard” for measuring and testing the margin distances, which was done in lung cancer and esophageal cancer [10, 11]. However, most rectal pathological specimen studies of micro-foci distances mainly focused on the distance of distal microscopical intramural spread (MIS) for surgical excision clearance. Few have measured the micro disease in the mesorectum and micro spread above the tumor [12]. None of them had incorporated both the retraction factors in the specimen surgical “move out” and pathological processing into correction. Accordingly, there is presently no consensus on the CTV margin for boost high-dose radiotherapy in rectal cancer, which is usually 1.5–2 cm in peripheral and 2–3 cm in superior and inferior directions or includes the whole mesorectal region [13–15]. This leads to massive unnecessary high-dose radiation to normal organs.

In this study, we measured the distances between tumor micro-foci and the gross tumor in the lateral (peripheral), inferior, and superior sides in rectal cancer pathological specimens. The retraction factors during operation and pathological processing had been calculated. The distances of in vivo tumor micro-foci were obtained after the correction of both retraction factors, which provided reference data for CTV margin distances of high-dose radiotherapy for rectal cancer.

## Materials and methods

### Clinical information

Twenty-eight patients with rectal cancer who received TME surgery at the Hunan Cancer Hospital from October 2016 to April 2017 were selected. Inclusion criteria: (1) patients with MRI-based clinical stage ≥ T3; (2) patients who refuse to receive radiotherapy and chemotherapy before surgery; (3) all patients who underwent endoscopic biopsy and adenocarcinoma was pathologically confirmed before surgery (which was referred to as “rectal cancer” in this paper); (4) patients for whom distant metastasis was not indicated through chest and abdominal CT and bone scintigraphy examination. Those 28 patient pathological specimens were retrospectively measured in 2022. This study was approved by the Hunan Cancer Hospital Ethics Committe and was performed in accordance with the ethical standards as laid down in the 1964 Declaration of Helsinki.

### Acquisition of preoperative image

All MRI examinations were performed using a 1.5-Tesla MRI scanner (Optima^®^MR 360; GE Medical Systems, Milwaukee, WI, USA) using a phased-array body coil. The patients were placed in the supine position, and preoperative pelvic enhanced standard MRI sequences were performed on all patients within 1 week of operation. Perpendicular to the rectal axis, the MRI scan spacing ranges from 0.3 to 0.5 mm, and scan thickness is 3 mm. The gross tumor volume (GTV) of the rectal cancer was delineated according to abnormal signals on T2WI. The delineation and thickness measurements were performed by an imaging diagnostic physician with more than 10 years of experience (L.W.).

### Processing of pathological specimens

**Fig. 1:**
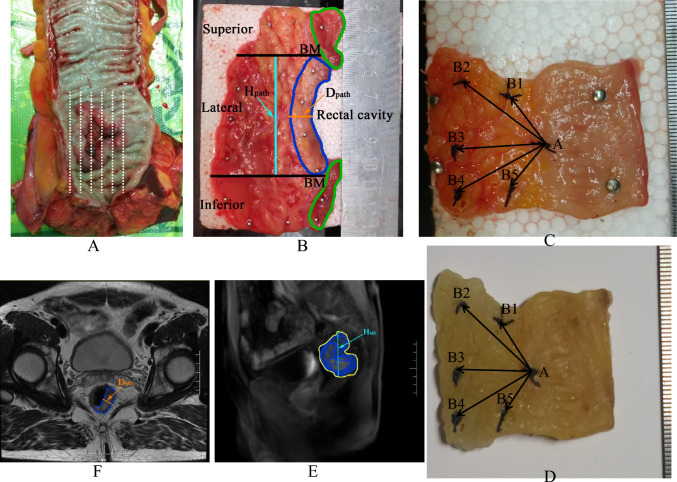
Processing of pathological specimens and measuring tumor retraction factors. **A** Immediate surgical specimen was cut vertically along the opposite side of the main tumor body, and then the tumor was evenly cut into tissue slices of 5 mm in thickness. **B** The middle piece of tissue slice was fixed to a foam plate with pins, the gross tumor range was mapped by naked eyes and the tumor depth and height were measured (marked as *D*_path_ and *H*_path_). **C** The adjacent 5 mm tissue slices were fixed to the foam plate similarly, then a surgical knot was tied at the midpoint of the mesangial side of the tumor and labeled as the standard point A, and several knot reference points B_1_, B_2_, B_3_, B_4_ and B_5_ were set in different directions of the mesentery. **D** After 24 h formalin dehydration. **E** The gross tumor was outlined on all layers of sagittal T2WI and was superimposed in the middle image. The tumor height in MRI was the vertical distance between the top and bottom tips of the outlines and marked as *H*_MR._ **F** the corresponding layer of MRI T2WI images at the widest part of the tumor was marked as *D*_MR_. BM: basis of measurement

TME surgery was conducted on all 28 patients in this study. Immediately after operation, processing of pathological specimens and measuring tumor retraction factors are illustrated and described in Fig. 1. This process was used to measure and calculate the retraction factor of specimen processing.

### Calculation of tumor retraction factors

Tumor deformation and retraction occurred during the process of TME surgery and postoperative pathological section preparation. The calculation of the retraction factor was divided into two steps: after the in vivo tumor was isolated, the first retraction occurred from the lack of pelvic fascia tractions, which was defined as the “in vivo–in vitro” retraction factor (*R*_1_); in the process of pathological specimen preparation, the in vitro tumor shrunk for the second time because of tissue dehydration, which was defined as the specimen processing retraction factor (*R*_2_).

#### Calculation of *R*_1_

The middle tissue slices of immediate surgical rectal cancer specimens (Fig. 1B) were obtained and fixed on a foam plate, as described above. The gross tumor range was mapped by the naked eye. To calculate the retraction factor in the lateral (peripheral) direction, the widest thickness was measured, which was marked as *D*_path_. The MRI T2WI images of all tumor layers were obtained, and the corresponding layer at the widest part of the tumor was selected (Fig. 1F), which was marked as *D*_MR_. The *R*_1_ value in the lateral direction was calculated using the following formula: *R*_1-L_ = *D*_path_/*D*_MR_. To calculate the retraction factor in the superior/inferior direction, the vertical height of the gross tumor in the specimen was measured and marked as *H*_path_ (Fig. 1B). The gross tumor was outlined on all layers of sagittal T2WI and superimposed in the middle image. The tumor height in the MRI was the vertical distance between the top and bottom tips of the outlines and marked as H_MR_ (Fig. 1E). The *R*_1_ value in the superior/inferior direction was calculated using the following formula: *R*_1-S/I_ = *H*_path_/*H*_MR_.

#### Calculation of *R*_2_

Because the degrees of tumor retraction varied in different directions, *R*_2_ was divided into three parts: inferior retraction (*R*_2-I_), lateral retraction (*R*_2-L_), and superior retraction (*R*_2-S_). The retraction factor (*R*_2_) of specimen processing was calculated using the changes of relative position between A and B_1_, B_2_, B_3_, B_4_, and B_5_ before dehydration (Fig. 1C) and after dehydration (Fig. 1D). AB_1-5_ represents the distance between A and the different B points. The formulas for calculating the retraction factors are as follows:

$$R_{2\text{-S}}=\frac{\text{AB}1\text{ after dehydration}}{\text{AB}1\text{ before dehydration}},$$

$$R_{2\text{-L}}= \left (\frac{\text{AB}2\text{ after dehydration}}{\text{AB}2\text{ before dehydration}}+\frac{\text{AB}3\text{ after dehydration}}{\text{AB}3\text{ before dehydration}}+\frac{\text{AB}4\text{ after dehydration}}{\text{AB}4\text{ before dehydration}} \right)/3$$

$$R_{2\text{-I}}=\frac{\text{AB}5\text{ after dehydration}}{\text{AB}5\text{ before dehydration}}.$$

### Measuring the distance of microcarcinoma

**Fig. 2:**
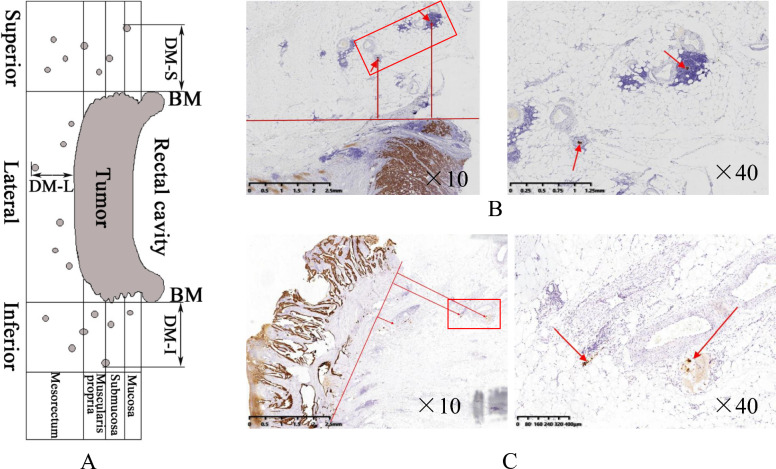
Measurement of micro-foci distance away from rectal gross tumor. **A** Schematic show of micro-foci distance measurement. **B** Micro-foci in the superior side. **C** Micro-foci in the lateral (peripheral) side. BM: basis of measurement; DM-I: distance of micro-foci inferiorly; DM-S: distance of micro-foci superiorly; DM-L: distance of micro-foci laterally

For pathological specimens, the middle 5 mm tissue specimens in 28 cases were divided into three paraffin tissues (separated by BM lines) of the lateral, inferior, and superior sides of the gross tumor (Fig. 2A). All paraffin tissues were sliced continuously at intervals of 2 mm, and each slice was 4 μm thick. The central three pieces were taken, with a total of 252 pieces taken for observation and examination of tumor micro-foci.

The sections were stained with eosin and methylene blue, and the whole section was first reviewed by a senior pathologist (X.Y.C.) using a low-power microscope (10×) to sketch the peripheral boundary of the primary gross tumor. If the edge of the primary focus showed a spiculated margin, it was sketched close to the root of spiculation. All tumor micro-foci located in the intramural and mesorectum were sketched on the pathological section. The distance from the outer edge of the gross tumor was measured, which was defined as the shortest linear extensive distance from the outboard edge of the cancer nest to the edge of the gross tumor. The tumor micro-foci were classified according to their morphologic type and their positions in the tumor nodule (lymph node metastasis within the mesorectum was not included), tumor budding, endovascular foci, endolymphatic foci, and neural sheath foci [16]. The actual measured distance between the micro-focus below and above the gross tumor was defined as the nearest linear distance between the micro-focus and BM, which was marked as DM-I/DM-S. The actual distance of the lateral micro-focus was defined as the nearest linear distance between the micro-focus and the primary gross tumor, which was recorded as DM-L (Fig. 2). After correction of the respective retraction factors, the in vivo distances of tumor micro-foci were recorded as DM-I_in vivo_, DM-S_in vivo_, and DM-L_in vivo_.

By measuring the DM-I, DM-S, and DM-L values and incorporating the *R*_1_ and *R*_2_ retraction factors in the respective directions, the distance of micro-foci in vivo was calculated as DM-L_in vivo_ = DM-L/(*R*_1-L_ × *R*_2-L_), DM-S_in vivo_ = DM-S/(*R*_1-S/I_ × *R*_2-S_), DM-I_in vivo_ = DM-I/(*R*_1-S/I_ × *R*_2-I_).

### Statistical analysis

SPSS 17.0 software was used for statistical analysis. The distances of lateral, inferior, and superior “in vivo” tumor micro-foci and 95% frequency value for 28 patients were calculated. Independent sample *t* tests and variance analysis were carried out. Numerical and categorical variables were compared using Chi-square and Student’s *t* tests. Differences were considered statistically significant for *P* values ≤ 0.05.

## Results

### Clinical characteristics of the patients

Overall, 28 patients were included in this study. Their characteristics are summarized in Table 1. The patients included 17 males (60.7%) and 11 females (39.3%), with an average age of 61.9 (41–80) years. Tumor distribution was governed by middle and low rectal cancers (19 cases). Tumor differentiation was mainly moderate (24 cases), while there were 13 cases with parenteral lymph node metastasis (46.4%).

**Table 1:** Characteristics of patients and tumor

	*N* (%)
Patients	28
Age (years)	
≥ 60	18 (64.3)
< 60	10 (35.7)
Gender	
Male	17 (60.7)
Female	11 (39.3)
Pathology	
High differentiation	1 (3.6)
Moderate differentiation	24 (85.7)
Poor differentiation	3 (10.7)
Pathological T stage	
T2	5 (17.9)
T3	23 (82.1)
Pathological N stage	
N0	15 (53.6)
N1	10 (35.7)
N2	3 (10.7)
Stage	
II	15 (53.6)
III	13 (46.4)
Distance to the anal verge (cm)	
≤ 5	5 (17.9)
> 5, ≤ 10	14 (50.0)
> 10	9 (32.1)

### Retraction rates

The “in vivo–in vitro” retraction rate (*R*_1_) and the “pathological processing” retraction rate (*R*_2_) of the rectal specimens in three directions are summarized in Table 2. In the lateral direction, the total retraction factor ranged from 0.600 to 0.834, with a mean value of 0.734 ± 0.057 and median of 0.748. In the superior direction, it ranged from 0.624 to 0.864, with a mean of 0.726 ± 0.064 and median of 0.718. In the inferior direction, it ranged from 0.605 to 0.901, with a mean of 0.750 ± 0.066 and a median of 0.753.

**Table 2:** The surgical move out and pathological processing retraction factors of 28 rectal cancer patients

	Range	Mean	Median
R1			
R_1-L_	0.808–0.950	0.913 ± 0.034	0.921
R_1-S/I_	0.882–0.957	0.920 ± 0.021	0.921
R2			
R_2-L_	0.700–0.909	0.804 ± 0.051	0.826
R_2-S_	0.673–0.941	0.789 ± 0.066	0.811
R_2-I_	0.654–0.973	0.815 ± 0.071	0.783
R1 × R2			
Lateral	0.600–0.834	0.734 ± 0.057	0.748
Superior	0.624–0.864	0.726 ± 0.064	0.718
Inferior	0.605–0.901	0.750 ± 0.066	0.753

R1: “in vivo–in vitro” surgical move out retraction factor; R2: pathological processing retraction factor; R_1-L_: R1 in the lateral (circumferential) direction; R_1-S/I_: R1 in the Superior/Inferior direction; R_2-L_: R2 in the lateral (circumferential) direction; R_2-S_: R2 in the superior direction; R_2-I_: R2 in the inferior direction

### Statistics of tumor micro-foci distance of "in vivo" micro-foci

Of 28 patients, 14 cases (50.0%) were observed to have lateral micro-foci, eight cases (28.6%) were observed to have inferior micro-foci, and seven cases (25.0%) were observed to have superior micro-foci. There were 24 lateral tumor micro-foci. After correction with retraction factors, the minimum, maximum, median, and mean distances were 1.3 mm, 8.45 mm, 2.63 mm, and 3.19 mm, respectively. The frequency table data (Table 3) demonstrate that the 95% frequency value of the invasion range of lateral tumor micro-foci was within 6.44 mm for 28 patients. There were 13 inferior tumor micro-foci. After correction with retraction factors, the minimum, maximum, median, and mean distances were 1.22 mm, 9.47 mm, 2.60 mm, and 3.24 mm, respectively. The 95% frequency value of the invasion range of the inferior tumor micro-foci was within 5.54 mm for 28 patients (Table 4). There were 19 superior micro-loci. After correction with retraction factors, the minimum, maximum, median, and mean distances were 1.38 mm, 6.25 mm, 3.39 mm, and 3.37 mm, respectively. The 95% frequency value of the invasion range of the superior tumor micro-foci was within 5.42 mm for 28 patients (Table 5). The margins needed to treat 95% of the 28 rectal cancer patients in the lateral, inferior, and superior directions were 6.44 mm, 5.54 mm, and 5.42 mm, respectively.

**Table 3:** Lateral (peripheral) micro-foci distribution for 28 cases of rectal cancer

Micro-foci (mm)	Number	Cumulative	% number	Cumulative %
0	14	14	36.84	36.84
1.30	1	15	2.63	39.47
1.47	1	16	2.63	42.11
1.56	1	17	2.63	44.74
1.76	1	18	2.63	47.37
1.81	1	19	2.63	50.00
1.93	1	20	2.63	52.63
2.04	1	21	2.63	55.26
2.08	1	22	2.63	57.89
2.10	1	23	2.63	60.53
2.21	1	24	2.63	63.16
2.60	1	25	2.63	65.79
2.63	1	26	2.63	68.42
2.93	1	27	2.63	71.05
3.09	2	29	5.26	76.32
3.19	1	30	2.63	78.95
3.52	1	31	2.63	81.58
3.57	1	32	2.63	84.21
3.84	1	33	2.63	86.84
4.20	1	34	2.63	89.47
5.11	1	35	2.63	92.11
5.71	1	36	2.63	94.74
6.44	1	37	2.63	97.37
8.45	1	38	2.63	100.00

**Table 4:** Inferior micro-foci distribution for 28 cases of rectal cancer

Micro-foci (mm)	Number	Cumulative	% number	Cumulative %
0	20	20	60.61	60.61
1.22	1	21	3.03	63.64
1.43	1	22	3.03	66.67
1.46	1	23	3.03	69.70
1.66	1	24	3.03	72.73
1.67	1	25	3.03	75.76
2.00	1	26	3.03	78.79
2.60	1	27	3.03	81.82
2.80	1	28	3.03	84.85
3.33	1	29	3.03	87.88
3.49	1	30	3.03	90.91
5.40	1	31	3.03	93.94
5.54	1	32	3.03	96.97
9.47	1	33	3.03	100.00

**Table 5:** Superior micro-foci distribution for 28 cases of rectal cancer

Micro-foci (mm)	Number	Cumulative	% number	Cumulative %
0	21	21	52.50	52.50
1.38	1	22	2.50	55.00
1.54	1	23	2.50	57.50
1.71	1	24	2.50	60.00
1.79	1	25	2.50	62.50
1.92	1	26	2.50	65.00
2.07	1	27	2.50	67.50
2.52	1	28	2.50	70.00
2.64	1	29	2.50	72.50
3.24	1	30	2.50	75.00
3.39	1	31	2.50	77.50
3.46	1	32	2.50	80.00
3.54	1	33	2.50	82.50
3.78	1	34	2.50	85.00
3.86	1	35	2.50	87.50
4.66	1	36	2.50	90.00
4.83	1	37	2.50	92.50
5.42	1	38	2.50	95.00
5.96	1	39	2.50	97.50
6.25	1	40	2.50	100.00

## Discussion

A variety of CTV (or planning target volume, PTV) margins have been used in clinical trials of high-dose boost radiotherapy for rectal cancer. Appelt et al. defined 60 Gy CTV margins as GTV plus whole rectum at the same level [13]. In the UK EXPERT trial, the PTV_52Gy was created by adding a margin of 10 to 30 mm around the GTV, depending on the site and fixity [14]. In the EXPERT-C phase II clinical trial, the boost 5.4 Gy was prescribed to the assessable tumor with a 2 cm margin in all directions [15]. The ongoing clinical trial of RECTAL BOOST study protocol has not added any margin around the GTV [17]. Currently, there is no consensus on how much CTV margin around the GTV should be added to escalate to a higher radiation dose for treating rectal cancer.

Measuring the distance between subclinical disease and GTV in pathological specimens is the best way to accurately determine the CTV margin. However, there are insufficient studies on the distribution of micro-foci around the gross tumors of rectal cancer. Verrijssen et al. performed a meta-analysis on individual patient data of microscopic intramural extension of rectal cancer after neoadjuvant chemoradiation [12]. They concluded that a 5.5 mm margin was needed to include MIS for 95% of patients. However, this analysis only retrieved four individual datasets from 11 relevant studies. Three out of four patient populations only had distal MIS parallel to the bowel wall (for surgical purpose) analyzed, leaving the degree of micro spread foci in the circumferential mesorectum and superior bowel wall unknown. This is also relevant in the CTV margin of a radiation boost. Furthermore, the “in vivo–in vitro” deformation and pathological specimen fixation and processing shrinkage were not considered in their report. This would underestimate MIS to an unknown degree. Goldstein et al. studied 26 cases of sigmoid and rectum specimens and found longitudinal normal bowel shrinkages of up to 40% after surgical move out 10–20 min later. Further shrinkages of up to 57% after 12–18 h of 10% formalin fixation were observed [18]. Macroscopic tumor shrinkages were not reported in that study. Literature on other tumors, such as lung cancer and renal cancer, reported approximately 5% diameter shrinkages after fixation [19, 20]. In our study, surgical removal resulted in mean tumor shrinkages of 8% longitudinally and 8.7% perpendicularly, followed by 18.5% inferior, 21.1% superior, and 19.6% perpendicular shrinkages after formalin fixation (Table 2). Our results emphasize that rectal tumor retractions following both “in vivo–in vitro” surgical removal and specimen processing should be considered to correct the measured micro-foci margin. Not doing so will cause the margin distance to be underestimated. Besides the distal intramural spread, few studies have reported the micro-foci spread in the mesorectum. Guedj et al. found no distal mesorectal spread after NCRT, and more than 90% of mesorectal metastatic lymph node and extra-node micro-foci were around and within 3 cm above the tumor [21]. Shimada et al. found 8.1% (31/381) distal mesorectal spread in their study without NCRT [22]. Our study demonstrates that 50.0% (14/28) of cases had micro-foci circumferentially, and 28.6% (8/28) and 25.0% (7/28) of cases had inferior and superior micro-foci spread respectively. This included both mesorectal and intramural spread. NCRT had not been implemented before operation in our cases. The micro-foci distribution pattern and margin distance were not influenced by chemoradiation. Smith et al. reported that NCRT could result in tumor downsizing and downstaging, with 80% (36/45) having a fragmentation response pattern. Hence, more patients had micro-foci present [23]. However, a meta-analysis reported that 80% of NCRT-treated patients had concentric shrinkage without micro-foci [12]. The micro-foci distances reported in those studies had mean values of within 10 mm after NCRT without shrinkage correction. With front-line surgery, our study found that 19/28 (67.9%) cases had circumferential micro-foci, and only 4/28 (14.3%) and 4/28 (14.3%) cases had inferior and superior micro-foci, respectively. The mean circumferential distance was 3.20 mm, with a mean inferior distance of 3.24 mm and mean superior distance of 3.37 mm. Our data for these parameters are lower than most of those reported in the literature. One possible reason for this observation is that NCRT was not performed in our cases, suggesting that a fragmented tumor pattern could not occur.

Because surgery immediately after NCRT of 50.4 Gy irradiation is not an option in clinical practice, the micro disease distribution in pathological specimens at this time point is impossible to measure. However, the micro disease in the pathological specimen 6–8 weeks post-NCRT would theoretically display the more radiation-resistant fractions of the disease. Hence, the micro-foci distances measured at this time point will be more meaningful to set late course boost radiation margins. Currently, the micro-foci distance 6–8 weeks post-NCRT for LARC is under investigation by our team.

This study was still insufficient in measuring the “in vitro” retraction of rectal specimens. There is a possible mismatch error between the image and tumor layer measured on pathological specimens because of the thickness and spacing of MRI scans, which led to a tiny deviation in the final measured microinvasion distance. In addition, Even though our inclusion criteria were both clinical T3 and T4 cases, however, consequently there are no pathological T4 cases in this pilot study. There were potentially larger micro-foci distances around pathological T4 cases. The number of cases observed here and the number of surgical sections analyzed are still limited, especially for the number of micro-foci observed at the superior and inferior ends of the rectal cancer. Therefore, the 95% probability coverage distance between the superior and inferior ends of rectal cancer should be cautiously regarded. Because of logistical reasons, the whole-mount cross-sectional specimen examinations were not performed in this study. Some micro-foci could potentially have been missed. Even though total 252 pieces of 4 μm-thick slice had been studied, the percentage of histologically studied sections might be much less than 0.2% of the whole paraffin tissues. Most micro-foci might have been missed in this study. However, the most central 5 mm-thick tumor slices that included both the superior and inferior 3 cm normal bowel would minimally miss the furthest most micro-foci. The micro-foci distance could only be measured in 2D; however due to the irregularity of tumor infiltration growth, especially the winding growth in 3D real situation, there was the possibility that some tips of tissue of GTV would be misregarded as micro-foci in 2D measurement.

In summary, through the measurement of pathological specimens and correction of retractions, this study reports that the 95% coverage of the invasion area of peripheral tumor micro-foci in 28 patients with LARC was 6.44 mm. Additionally, this value was 5.54 mm and 5.42 mm for the inferior and superior micro-foci, respectively. Our results provide a reference for establishing the target area of high-dose radiotherapy for treating rectal cancer.

## Data Availability

The original data (including raw material data) are available upon request from Dr.Bao Xu-Jie, E-mail: baoxujie8@126.com.
